# Barriers to mental health service utilisation among medical students in Saudi Arabia

**DOI:** 10.3389/fpubh.2024.1371628

**Published:** 2024-04-12

**Authors:** Zaenb Alsalman, Marwa Mahmoud Shafey, Asma Al-Khofi, Jumana Alessa, Raghad Bukhamsin, May Bokhuwah, Ryhana Aljumaiah, Noura Al-makhaitah, Maryam Almaslami

**Affiliations:** ^1^Department of Family and Community Medicine, College of Medicine, King Faisal University, Al-Ahsa, Saudi Arabia; ^2^Department of Family and Community Medicine, College of Medicine, Imam Abdulrahman Bin Faisal University, Dammam, Saudi Arabia; ^3^College of Medicine, King Faisal University, Al-Ahsa, Saudi Arabia

**Keywords:** barrier, depression, medical student, mental health, service

## Abstract

**Introduction:**

Medical students experience high levels of stress, often due to academic demands, which can adversely affect their mental health. However, they frequently hesitate to seek and underutilise available mental health services. This study aimed to assess the perceived need for mental health services and identify the barriers to seeking help among undergraduate medical students.

**Materials and methods:**

This cross-sectional study recruited 480 undergraduate medical students from two main universities in the Eastern Province of Saudi Arabia. Data were collected through an online, self-administered questionnaire that encompassed sections on sociodemographic details, the Patient Health Questionnaire (PHQ-9), perceptions about the necessity for professional mental health care, service utilisation over the past year, and the Barriers to Access to Care Evaluation (BACE-III).

**Results:**

The study found that 33.6% of the participants showed signs of depression. Even though 42.5% expressed a perceived need for mental health services, only 16.2% actually utilised these services in the previous 12 months. In terms of barriers, attitudinal-related barriers received the highest mean score, followed by stigma- and instrumental-related barriers. Notably, students who had previously experienced academic failure and those who had sought mental health services were more inclined to report stigma- and instrumental-related barriers.

**Conclusion:**

Mental health challenges are notably prevalent among undergraduate medical students. Although there is a significant perceived need for professional mental health intervention, the actual utilisation rate remains low. The primary obstacles to seeking assistance are attitudinal and stigma-related barriers.

## Introduction

Mental disorders are among the leading causes of health-related burden worldwide (1). According to the World Health Organisation (WHO), one in every eight people globally suffered from a mental illness in 2019, with anxiety and depressive disorders being the most prevalent. In 2020, the COVID-19 pandemic increased the number of individuals living with anxiety and depressive disorders (2). Despite the availability of preventative and treatment options, a significant number of those with mental illnesses demonstrate low levels of help-seeking (2, 3). Additionally, many individuals who do seek mental health care fail to adhere to their treatment plans (3). According to the Saudi National Mental Health Survey, 20.2% of the Saudi population had at least one DSM-IV/CIDI disorder in the previous 12 months, with only 13.7% of these individuals seeking professional assistance (4, 5).

Students engaged in health profession disciplines face pronounced stress, often attributable to academic pressures, competition for higher grades, limited leisure opportunities, and other factors potentially detrimental to their mental well-being (6). The prevalence of depression, anxiety, burnout, and stress among medical students has steadily increased, leading to unfavourable consequences such as unprofessional behaviours, chronic health issues, diminished quality of life, and poor educational achievement. Therefore, these issues not only pose significant economic and societal challenges but also have potential ramifications for the quality of future patient care (3, 7). Despite being well-informed about mental health, many medical students are reluctant to seek out and utilise mental health services to address their personal challenges during their time in medical school (8, 9).

Over recent years, mental disorders among medical students have received considerable research attention, with numerous studies aimed at estimating the prevalence and risk factors of mental problems (7, 9, 10). Globally, around 12–50% of undergraduate university students have one or more mental health issues (11). Even though it is expected that medical students have better access to mental health services, help-seeking behaviour might be influenced by a variety of variables such as financial concerns, lack of awareness, difficulty in accessing services, and intrapersonal dilemma (12–15). Moreover, cultural factors such as family background, religion, and social norms have an impact on how interact with mental issues (12, 14, 16). Gaining a deeper insight into the disparity between the recognition of treatment needs and the actual provision of care can guide administrative strategies to better address students’ mental health concerns. In light of this, our study endeavoured to assess the perceived need for mental health services and identify the barriers hindering help-seeking among undergraduate medical students in the Eastern Province of Saudi Arabia.

## Materials and methods

### Setting and participants

A cross-sectional study was conducted among undergraduate medical students from two leading universities in the Eastern Province of Saudi Arabia, namely King Faisal University (KFU) and Imam Abdulrahman Bin Faisal University (IAU).

As no previous studies have explored the perceived need for mental health services in Saudi Arabia among medical students, the sample size was calculated assuming a perceived need of 50%. With a 95% confidence interval, a 0.05 margin of error, and 80% power, the minimum sample size was estimated at 384 using Epi Info software (version 3.4.3). To enhance the response rate, the estimated number was increased by 20%, targeting a total of 460 participants. A comprehensive list of students from the second to sixth years at both universities was acquired from the academic affairs department. Participants were selected using a simple random sampling technique, with the sample size proportionally distributed based on the number of students at each university’s medical college (45% from KFU and 55% from IAU).

The study was approved by the Ethical Committee of King Faisal University, Al Ahsa, Saudi Arabia (Ref. No: KFU-REC-2022-MAY-ETHICS31; date: 24 May 2022). Additionally, participants received a brief description of the study’s purpose and a link to a non-personalised consent form.

### Instruments

An anonymous online questionnaire was distributed via email to medical students. The questionnaire was divided into four sections. The first section covered sociodemographic data such as age, gender, living conditions (with family, in a university dormitory, or alone), previous academic failures, personal and family histories of psychiatric illnesses, and the use of psychiatric medications. The second section incorporated the Patient Health Questionnaire (PHQ-9), which consists of nine statements, each with four possible responses ranging from 0 to 3. The sum of these responses provides a total score ranging from 0 to 27; the scores are interpreted as follows: 0–4 (minimal), 5–9 (mild), 10–14 (moderate), 15–19 (moderately severe), and 20 or more (severe) (17, 18). Additionally, scores below 10 have shown 88% sensitivity and specificity in diagnosing major depression (17). Therefore, scores of 10 or lower were perceived as non-indicative of depression, while scores exceeding 10 were considered indicative of depression. Section 3 delved into the students’ perceived necessity for professional mental health care and their engagement with these services over the preceding 12 months. The fourth section assessed barriers to the utilisation of mental health services using the Barriers to Access to Care Evaluation (BACE-III); however, only those students who perceived a need for mental health support were prompted to complete the BACE-III questionnaire (19). The standard BACE-III consists of 30 items, divided into three barrier subscales: 12 items for stigma-related barriers, 10 for attitudinal-related barriers, and 8 for instrumental-related barriers. Responses are scored from 0 (not at all) to 3 (a lot), with higher scores indicating greater perceived barriers. Five of the original 30 items include a fifth response option, ‘Not applicable’. However, these were excluded from this study as they were not deemed relevant for the student cohort. Consequently, the adapted version included 25 items, with the three subscales adjusted to eight items for stigma-related barriers, 10 for attitudinal barriers, and seven for instrumental barriers. The questionnaire was reviewed by three experts in the field of family medicine as well as mental health and piloted among 30 undergraduate students from different health-related fields outside the sample to test its validity and reliability. The BACE-III instrument reliability (internal consistency was 0.852; for stigma sub-scale: 0.841; attitudinal sub-scale: 0.673 and instrumental sub-scale: 0.689).

### Data analysis

The collected data were analysed using the Statistical Package for the Social Sciences (SPSS) version 25. For descriptive statistics, frequencies and percentages were used for categorical variables, while means and standard deviations were used for continuous variables. Results for each barrier item were presented in three ways, according to the BACE-III scale: the mean score for the item, ‘barrier to any degree’ (the percentage of respondents answering 1, 2, or 3), and ‘major barrier’ (the percentage of respondents answering 3) (19). The mean of each subscale was also calculated.

Differences in the pattern of mental health service utilisation (categorical) were assessed in relation to students’ characteristics (categorical) using chi-square test. Furthermore, differences in the mean scores of the BACE-III barrier subscales (continuous data) were assessed in relation to students’ characteristics using the independent *t*-test and one-way ANOVA for normally distributed data (attitudinal subscale), and the Mann–Whitney U and Kruskal-Wallis tests for non-normally distributed continuous data (stigma and instrumental subscales). Additionally, to examine the relationship between demographic variables and the mean score of the BACE-III subscales, both univariate and multiple linear regression models were employed. A *p* value of <0.05 was considered statistically significant.

## Results

### Characteristics of the participants

A total of 480 students (87% response rate) participated in the study, with a mean age of 21.40 years (*SD* = 1.86). The majority of the students were female (69.4%), single (87.5%), and lived with their families (82.3%). Only 12.3% of the students reported a personal history of mental illness: anxiety (7.1%, *n* = 34), depression (6.3%, *n* = 30), schizophrenia (0.4%, *n* = 2), and bipolar disorder (0.6%, *n* = 3). Additionally, 20.4% of students had a family history of mental illness. Furthermore, 40.6% of the students were unaware of the availability of mental health counselling services in their colleges. The remaining sociodemographic characteristics are presented in Table 1.

**Table 1:** Sociodemographic and medical characteristics of the medical students (*n* = 480).

Variables		No (%)
Age in years	Range (Mean ± SD)	18–27 (21.4 ± 1.9)
Gender	Male	147 (30.6)
	Female	333 (69.4)
Marital status	Married	55 (11.5)
	Single	420 (87.5)
	Divorced	5 (1.0)
Academic year	2nd	57 (11.8)
	3rd	66 (13.8)
	4th	141 (29.4)
	5th	94 (19.6)
	6th	122 (25.4)
Living circumstances	With family	395 (82.3)
	On campus (Dorms)	52 (10.8)
	Alone	33 (6.9)
History of academic failure	No	381 (79.4)
	Yes	99 (20.6)
Personal history of psychiatric illness	No	421 (87.7)
	Yes	59 (12.3)
Self-reported psychiatric illness	Anxiety	34 (7.1)
	Depression	30 (6.3)
	Schizophrenia	2 (0.4)
	Bipolar disorder	3 (0.6)
Family history of psychiatric illness	No	382 (79.6)
	Yes	98 (20.4)
Self-reported family psychiatric illness	Depression	46 (9.6)
	Anxiety	23 (4.8)
	Bipolar disorder	10 (2.1)
	Schizophrenia	17 (3.5)
	PD	1 (0.2)
History of taking psychiatric medications	Never	443 (92.3)
	Ever user	37 (7.7)
Awareness about mental health counselling services in your university	Yes	285 (59.4)
	No	195 (40.6)

### Depression prevalence

**Figure 1:**
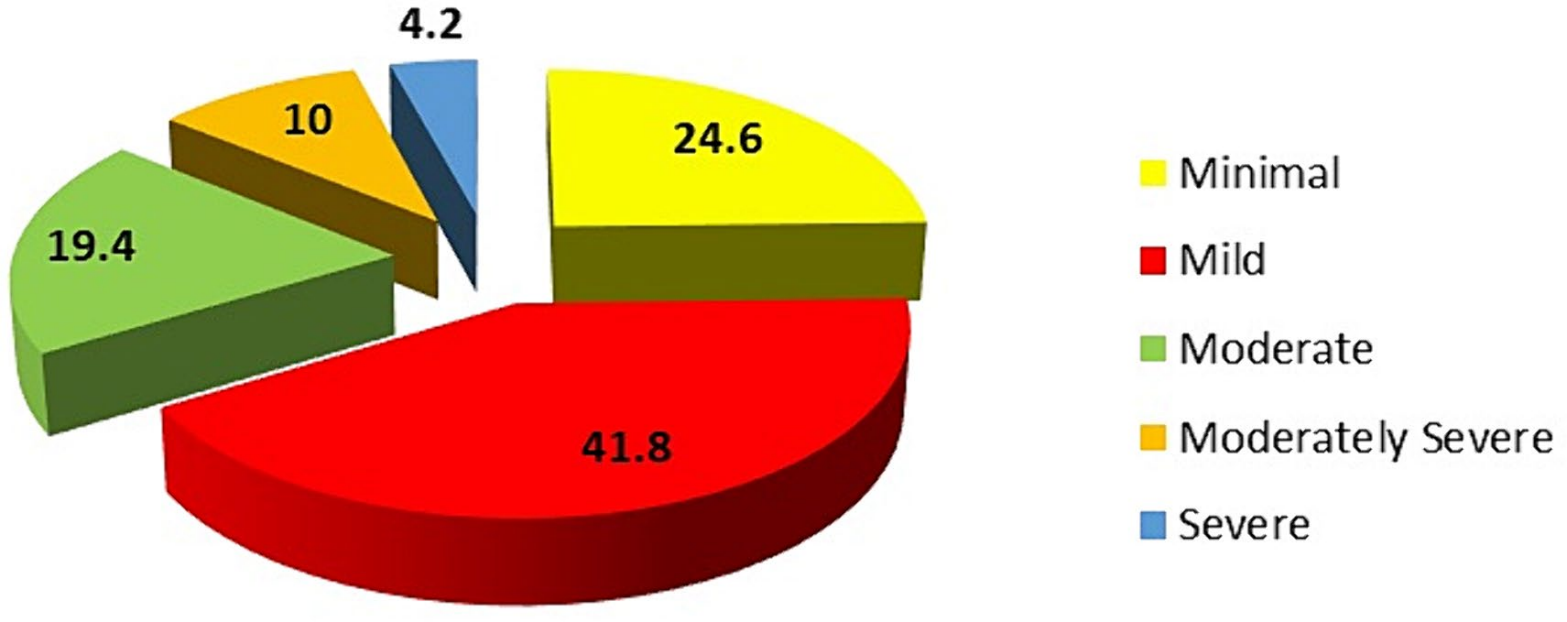
Distribution of students according to the Patient Health Questionnaire Depression Score (PHQ-9).

Based on the PHQ-9 score, 24.2% of the students exhibited minimal depression, 41.8% had mild depression, 19.4% showed moderate depression, 10.0% experienced moderate to severe depression, and 4.2% had severe depression (Figure 1). The prevalence of depression, assessed using a PHQ-9 score of ≥10, was found to be 33.6%.

### Mental health services

**Figure 2:**
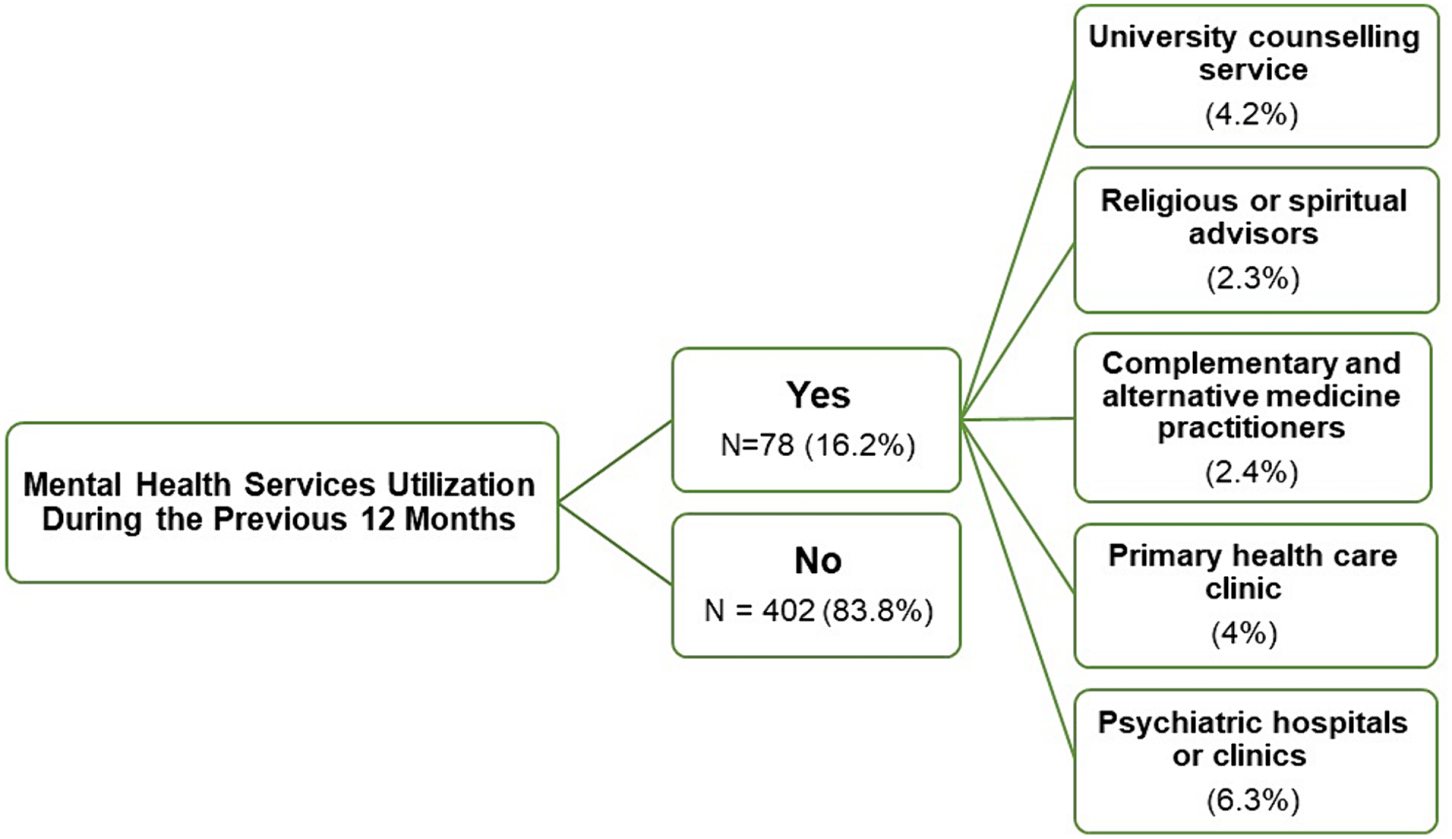
A flowchart representing the mental health services utilised by medical students in the previous 12 months.

The majority of students (83.8%, *n* = 402) reported that they had not utilised any mental health services in the past 12 months. In contrast, 16.2% (*n* = 78) of the students reported service utilisation during this period: 6.3% (*n* = 30) utilised psychiatric clinics, 4.2% (*n* = 20) accessed the university counselling service, and 4.0% (*n* = 19) visited primary care clinics (Figure 2). When examining factors related to the utilisation of mental health services, there was a significant association between utilisation and students living in university dormitories, those with a personal history of psychiatric illness, and students who had previously used psychiatric medications (*p* < 0.05, Table 2).

**Table 2:** Factors associated with utilisation and the perceived needs of mental health services (*n* = 480).

	Utilisation of mental health services *N* (%)			Perceived needs for counselling *N* (%)		
	No	Yes	*p*-value	No	Yes	*p*-value
	402 (83.8%)	78 (16.2%)		276 (57.5%)	204 (42.5%)	
Gender						
Male	119 (81.0)	28 (19.0)	0.270	98 (66.7)	49 (33.3)	0.007^*^
Female	283 (85.0)	50 (15.0)		178 (53.5)	155 (46.5)	
Living area						
With family	334 (84.6)	61 (16.4)		234 (59.2)	161 (40.8)	
On campus	37 (71.2)	15 (28.8)	0.012^*^	27 (51.9)	25 (48.1)	0.211
Alone	31 (93.9)	2 (6.1)		15 (45.5)	18 (54.5)	
Academic year						
2nd	51 (89.5)	6 (10.5)		36 (63.2)	21 (36.8)	
3rd	56 (84.8)	10 (15.2)	0.608	40 (60.6)	26 (39.4)	0.424
4th	115 (81.6)	26 (18.4)		73 (51.8)	68 (48.2)	
5th	76 (80.9)	18 (19.1)		52 (55.3)	42 (44.7)	
6th	104 (85.2)	184 (14.8)		75 (61.5)	47 (38.5)	
History of academic failure						
No	323 (84.8)	58 (15.2)	0.232	218 (57.2)	163 (42.8)	0.806
Yes	79 (79.8)	20 (20.2)		58 (58.6)	41 (41.4)	
Personal history of psychiatric illness						
No	377 (89.5)	44 (10.5)	<0.001	254 (60.3)	167 (39.7)	0.001^*^
Yes	25 (42.4)	34 (57.6)		22 (37.3)	37 (62.7)	
Family history of psychiatric illness						
No	326 (85.3)	56 (14.7)	0.062	227 (59.4)	155 (40.6)	0.092
Yes	76 (77.6)	22 (22.4)		49 (50.0)	49 (50.0)	
History of taking psychiatric medications						
Never	391 (88.3)	52 (11.7)	<0.001	260 (58.7)	183 (41.3)	0.068
Ever user	11 (29.7)	26 (70.3)		16 (43.2)	21 (56.8)	
Depression score						
Minimal	106 (89.8)	12 (10.2)		93 (78.8)	25 (21.2)	
Mild	176 (87.6)	25 (12.4)	<0.001	124 (61.7)	77 (38.3)	<0.001
Moderate	74 (79.6)	19 (20.4)		37 (39.8)	56 (60.2)	
Moderately severe	37 (77.1)	11 (22.9)		18 (37.5)	30 (62.5)	
Severe	9 (45.0)	11 (55.0)		4 (20.0)	16 (80.0)	

^*^p < 0.05 (significant).

Out of the total participants, 42.5% (*n* = 204) reported a perceived need for mental services. There was a statistically significant association between the perception of need and both gender and a personal history of psychiatric illness. Female students and those with a personal history of psychiatric illness were more likely to perceive a need for mental health services (*p* < 0.05). Moreover, students with severe depression exhibited a higher demand for and utilisation of mental health services compared to those with milder cases (*p* < 0.05, Table 2).

### Barriers to utilising mental health services

Table 3 presents the barriers to mental health service utilisation among students who perceived a need for such services in the last 12 months (*n* = 204). Based on the average barrier scores, the primary reason for not seeking treatment was ‘Wanted to solve the problem on my own’, reported by 89.7% as a barrier to any degree and 47.1% as a major barrier. The second most common barrier was ‘Thinking the problem would get better by itself’, reported by 80.4% as a barrier to any degree and 32.4% as a major barrier. Ranking third was a ‘Dislike of talking about feelings, emotions, or thoughts’ identified by 80.9% as a barrier to any degree and 29.4% as a major barrier. The fourth barrier was ‘Difficulty taking time off from education’, with 77.9% considering it a barrier to any degree and 29.9% viewing it as a major barrier. The fifth most cited reason was ‘Being unsure where to go to get professional care’, reported by 73.0% as a barrier to any degree and 27.5% as a major barrier.

**Table 3:** Barriers to receiving professional mental health care among students who perceived the need for mental health service in the past 12 months (*n* = 204).

Barriers	Barrier to any degree	Major barrier	Mean (SD)	Rank
	No (%)	No (%)		
Stigma-related barriers				
Concern that I might be seen as weak for having a mental health problem	117 (57.4)	24 (11.8)	1.00 (1.05)	15
Concern about what my family might think, say, do or feel	141 (69.1)	51 (25.0)	1.39 (1.17)	6
Feeling embarrassed or ashamed	106 (52.0)	24 (11.8)	0.90 (1.04)	18
Concern that I might be seen as ‘crazy’	78 (38.2)	16 (7.8)	0.63 (0.94)	21
Concern that people I know might find out	111 (54.4)	33 (16.2)	0.99 (1.11)	16
Concern that people might not take me seriously if they found out I was having professional care	111 (54.4)	34 (16.7)	1.08 (1.15)	13
Not wanting a mental health problem to be on my medical records	124 (60.8)	43 (21.1)	1.22 (1.18)	9
Concern about what my friends might think, say or do	78 (38.2)	11 (5.4)	0.60 (0.89)	22
Stigma-related barriers Mean Score(±SD)/Median/Range	7.79 (±5.89)/7 0–24			
Attitudinal-related barriers				
Wanting to solve the problem on my own	183 (89.7)	96 (47.1)	2.09 (1.03)	1
Fear of being put in hospital against my will	62 (30.4)	17 (8.3)	0.55 (0.95)	23
Thinking the problem would get better by itself	164 (80.4)	66 (32.4)	1.72 (1.12)	2
Preferring to get alternative forms of care (e.g., traditional/religious healing or alternative/complementary therapies)	112 (54.9)	25 (12.3)	0.96 (1.05)	17
Thinking that professional care probably would not help	135 (66.2)	33 (16.2)	1.17 (1.07)	10
Dislike of talking about my feelings, emotions, or thoughts	165 (80.9)	60 (29.4)	1.66 (1.2)	3
Concerns about the treatments available (e.g., medication side effects)	133 (65.2)	41 (20.1)	1.31 (1.15)	7
Having had previous bad experiences with professional care for mental health	57 (27.9)	11 (5.4)	0.49 (0.879)	25
Preferring to get help from family or friends	116 (56.9)	30 (14.7)	1.01 (1.09)	14
Thinking I did not have a problem	124 (60.8)	33 (16.2)	1.09 (1.2)	12
Attitudinal-related barriers Mean Score(±SD)/Median/Range	12.05 (±5.31)/12 0–27			
Instrumental-related barriers (accessibility)				
Being unsure where to go to get professional care	149 (73.0)	56 (27.5)	1.49 (1.16)	5
Problems with transport or travelling to appointments	86 (42.2)	24 (11.8)	0.77 (1.06)	20
Not being able to afford the financial costs involved	133 (65.2)	66 (32.4)	1.17 (1.07)	11
Professionals from my own ethnic or cultural group not being available	67 (32.8)	11 (5.4)	0.55 (0.90)	24
Being too unwell to ask for help	97 (47.5)	27 (13.2)	0.90 (1.98)	19
Difficulty taking time off education	159 (77.9)	61 (29.9)	1.57 (1.14)	4
Having no one who could help me get professional care	133 (65.2)	40 (19.6)	1.25 (1.13)	8
Instrumental-related barriers Mean Score(±SD)/ Median/Range	7.22 (±4.06)/12 0–17			

In terms of barrier subscales, the attitudinal-related barriers had the highest average score compared to the other subscales (attitudinal barrier: 12.05 [± 5.31], stigma-related barrier: 7.79 [± 5.89], and instrumental-related barrier: 7.22 [± 4.06]).

Table 4 shows the association between barrier subscales and students’ characteristics. Students who had previously experienced academic failure, as well as those who had utilised mental health services in the past, exhibited higher barrier scores. Notably, there were statistically significant differences in the stigma- and instrumental-related subscales (*p* < 0.05).

**Table 4:** Differences in BACE-III barrier subscales (Mean ± SD) in regards to different students’ characteristics (*n* = 204).

Variable	BACE-III barrier subscales		
	Stigma related barriers	Attitudinal related barriers	Instrumental related barriers
	Mean (SD)	Mean (SD)	Mean (SD)
	7.79 (±5.89)	12.05 (±5.31)	7.22 (±4.06)
Gender			
Male (49)	9.06 ± 6.22	13.16 ± 5.17	7.28 ± 4.03
Female(155)	7.40 ± 5.75	11.70 ± 3.33	7.20 ± 4.09
*p* value	0.091	0.094	0.879
Living area			
With family (161)	7.82 ± 5.99	12.13 ± 5.32	7.02 ± 3.90
On campus (25)	8.64 ± 5.93	11.32 ± 4.95	8.28 ± 5.02
Alone (18)	6.44 ± 4.90	12.33 ± 5.97	7.55 ± 4.08
*p* value	0.545	0.755	0.351
Academic year			
2nd	7.14 ± 5.42	12.87 ± 7.18	6.76 ± 4.17
3rd	8.00 ± 5.32	10.73 ± 4.71	8.46 ± 3.95
4th	7.45 ± 5.78	11.81 ± 4.89	7.44 ± 4.01
5th	7.16 ± 6.29	13.17 ± 4.99	6.56 ± 3.90
6th	9.04 ± 6.21	11.78 ± 5.52	6.98 ± 4.32
*p* value	0.496	0.383	0.338
History of academic failure			
No (163)	7.30 ± 6.07	11.81 ± 5.24	6.88 ± 3.99
Yes (41)	9.78 ± 4.68	13.02 ± 5.56	8.56 ± 4.12
*p* value	0.002^*^	0.192	0.022^*^
Personal history of psychiatric illness			
No (167)	7.72 ± 5.88	12.24 ± 5.27	7.11 ± 4.07
Yes (37)	8.16 ± 5.97	11.27 ± 5.49	7.70 ± 4.05
*p* value	0.659	0.290	0.385
Family history of psychiatric illness			
No (155)	7.91 ± 5.83	12.58 ± 5.34	7.32 ± 4.03
Yes (49)	7.44 ± 6.13	11.02 ± 5.16	6.89 ± 4.21
*p* value	0.537	0.119	0.542
History of taking psychiatric medications			
Never (183)	7.55 ± 5.78	11.97 ± 5.33	7.70 ± 4.04
Ever user (21)	9.95 ± 6.48	12.76 ± 5.23	8.76 ± 4.12
*p* value	0.099	0.521	0.056
Ever utilised mental health services during the past 12 months			
Never (149)	7.16 ± 5.71	11.88 ± 5.07	6.60 ± 3.91
Yes (55)	9.52 ± 6.06	12.50 ± 5.94	8.89 ± 4.04
*p* value	0.012^*^	0.459	<0.001^*^

Differences regarding stigma and instrumental barriers have been analysed using Mann–Whitney and Kruskual Wallis test; meanwhile those for attitudinal related barriers were analysed using t test and ANOVA. ^*^Significant *p* value < 0.05.

### Predictors of mental health service utilisation barriers

The univariate analysis shows that students who had previously experienced academic failure were 2.480 times more likely to perceive stigma-related barriers. Similarly, those who had previously utilised mental health services were 2.366 times more likely to exhibit stigma-related barriers. However, after adjustments in the multivariate analysis, the sole predictor was the utilisation of mental health services. Specifically, students who had used these services were 2.042 times more likely to report stigma-related barriers (Table 5).

**Table 5:** Predictors of stigma related barriers to receiving professional mental health care in univariate and multivariable linear regression (*n* = 204).

Variable	Stigma related barriers			
	Univariate		Multivariate	
	β [CI:95%]	*p* value	β [CI:95%]	*p* value
Age	0.46 [−0.03,0.96]	0.065	0.21 [−0.33,0.75]	0.444
Gender (Ref: Male)	−1.66 [−3.56,0.23]	0.085	−0.84 [−2.84,1.15]	0.406
Female				
History of academic failure (Ref: No)	2.48 [0.47,4.48]	0.016^*^	−1.82 [−0.33,3.98]	0.097
Yes				
Personal history of psychiatric illness (Ref: No)	0.44 [−1.67,2.56]	0.680	−1.99 [−4.77,0.80]	0.162
Yes				
Family history of psychiatric illness (Ref: No)	−0.46 [−2.37,1.45]	0.634	−0.33 [−2.29, 1.62]	0.736
Yes				
History of taking psychiatric medications (Ref: Never)	2.44 [−0.26,5.06]	0.077	2.56 [−0.92,6.12]	0.147
Ever user				
Ever utilised mental health services during the past 12 months (Ref: No)	2.37 [0.56,4.17]	0.011^*^	2.04 [0.03,4.00]	0.047^*^
Yes				

^*^Significant *p* value < 0.05.

Univariate and multivariate analyses, as presented in Table 6, confirmed that none of the students’ characteristics predicted attitudinal-related barriers.

**Table 6:** Predictors of attitudinal related barriers to receiving professional mental health care in univariate and multivariable linear regression (*n* = 204).

Variable	Attitudinal related barriers			
	Univariate		Multivariate	
	β [CI:95%]	*p*-value	β [CI:95%]	*p*-value
Age	0.07 [−0.38, 0.51]	0.770	−0.12 [−0.62, 0.37]	0.621
Gender (Ref: Male)	−1.46 [−3.17, 0.25]	0.94	−1.39 [−3.2, 0.43]	0.134
Female				
History of academic failure (Ref: No)	0.07 [−0.52, 0.66]	0.814	1.08 [−0.90, 3.05]	0.284
Yes				
Personal history of psychiatric illness (Ref: No)	−1.02 [−2.93, 0.88]	0.290	−2.48 [−5.04, 0.07]	0.057
Yes				
Family history of psychiatric illness (Ref: No)	−1.36 [−3.07, 0.35]	0.119	−0.91 [−2.70, 0.88]	0.317
Yes				
History of taking psychiatric medications (Ref: Never)	0.52 [−1.63, 3.21]	0.789	2.48 [0.74, 5.70]	0.131
Ever user				
Ever utilised mental health services during the past 12 months (Ref: No)	0.62 [−1.03, 2.28]	0.459	0.61 [−1.23, −2.4]	0.511
Yes				

^*^Significant *p* value < 0.05.

In terms of instrumental-related barriers, univariate linear regression results indicate that students with prior academic failures were 1.678 times more likely to report these barriers. Additionally, those who had previously utilised mental health services were 2.287 times more likely to perceive instrumental-related barriers. After adjustments in the multivariate analysis, age emerged as a predictor. Specifically, for each 1-year decrease in age, there was an associated 0.373 increase in the likelihood of perceiving instrumental-related barriers. Students with a history of academic failure were 1.956 times more likely to perceive instrumental barriers, and those who had used mental health services were 2.194 times more likely to report them (Table 7).

**Table 7:** Predictors of Instrumental related barriers to receiving professional mental health care in univariate and multivariable linear regression (*n* = 204).

Variable	Instrumental related barriers			
	Univariate		Multivariate	
	β [CI:95%]	*p*-value	β [CI:95%]	*p*-value
Age	−0.19 [−0.53, 0.15]	0.275	−0.37 [−0.74, −0.00]	0.047^*^
Gender (Ref: Male)	−0.09 [−1.40, 1.23]	0.898	0.10 [−1.25, 1.45]	0.885
Female				
History of academic failure (Ref: No)	1.68 [0.29, 3.063]	0.018^*^	1.96 [0.49, 3.42]	0.009^*^
Yes				
Personal history of psychiatric illness (Ref: No)	0.59 [−0.87, 2.045]	0.427	−1.17 [−3.06, 0.72]	0.223
Yes				
Family history of psychiatric illness (Ref: No)	0.42 [−1.74, 0.89]	0.526	−0.22 [−1.54, 1.11]	0.748
Yes				
History of taking psychiatric medications (Ref: Never)	1.72 [−0.12, 3.56]	0.067	1.51 [−0.87, 3.89]	0.212
Ever user				
Ever utilised mental health services during the past 12 months (Ref: No)	2.29 [1.06, 3.52]	<0.001^*^	2.19 [0.83, 3.56]	0.002^*^
Yes				

^*^Significant *p* value < 0.05.

## Discussion

The prevalence of mental health disorders among undergraduate medical students is believed to be higher than in other individuals of comparable age in the general population (20). This heightened vulnerability is often attributed to the stress associated with studying medicine (21). In this study, the reported prevalence of depression (33%) was consistent with that reported in a meta-analysis study spanning 43 countries (20). However, the study by Ewid et al., which aimed to investigate the impact of the COVID-19 pandemic on the mental health of Saudi medical students, reported a slightly lower prevalence (23%) (22). The discrepancy between their findings and ours might be attributed to the fact that the majority of our participants were female, and studies have indicated that females are generally more susceptible to psychological distress (22).

Unmet mental health needs are believed to place a significant strain on community health and productivity (20). According to the current study, nearly 4 in 10 students reported a perceived need for professional mental health support. This perceived need was particularly pronounced among female students and those with a personal history of psychiatric illness. Such a high level of perceived need has been reported in previous studies (23, 24). Despite the high proportion of students reporting depressive symptoms and expressing a perceived need for help, these services remain underutilised. In this study, only 16.2% of students who felt a need for treatment sought professional help in the previous 12 months. Such utilisation rates, ranging from 18 to 23%, have also been observed among university students in recent studies conducted in Egypt and the United States (23, 25). It is worth noting that, in our study, students with severe depression were more likely to recognise a need for treatment and subsequently seek help compared to those with mild and moderate depression, which is consistent with previous literature (3, 26). This suggests that many students delay seeking care, even though early-stage cases are often easier to address (3).

Various factors could explain this unmet demand for help. However, in our study, the dominant barrier was attitudinal, with most students preferring to tackle their problems independently, hoping issues would naturally resolve, or feeling reluctant to share their emotions and thoughts. This observation is in line with a previous study that identified a preference for self-management as a significant obstacle to seeking formal mental health care among university students experiencing depression and anxiety (27). Additionally, our results underscore the prevailing hypothesis linking mental health literacy with tendencies towards help-seeking behaviours (3, 6). These findings emphasise the relevance of cultural diversity, which continues to have a crucial role in attitudes about mental disease treatment. In Saudi Arabia, a predominantly Muslim population country, a considerable number of people believe that mental illness is a result of insufficient religiosity and are hesitant to seek professional help, despite significant advancements in the mental health care system (15).

The stigma associated with mental health was the second most frequently reported obstacle in our research. In contrast, a study by Noorwali et al. indicated stigma as the principal factor deterring young Saudi adults from pursuing mental health services (14). These findings might be attributed to the persisting stigmatisation of mental illnesses in Arab nations, characterised by prevailing negative sentiments surrounding mental disorders and associated medical services (28). Furthermore, it is widely acknowledged that doctors and medical students are hesitant to engage in therapy, particularly for mental problems. In addition, it has been hypothesised that doctors stigmatise mental illness more than the general population because of the idea that seeking care for a mental condition may negatively affect one’s legal licence to practise medicine (9, 29).

Although our participants rated instrumental barriers the lowest overall, Egyptian medical students identified them as their most significant barrier (23). This discrepancy might be attributed to the relative accessibility of Saudi Arabia’s healthcare system, which offers cost-free psychiatric services (3). Even with the presence of qualified mental health mentors and a dedicated teaching referral clinic aimed at assisting distressed students, nearly 40% were unaware of these resources. These findings are consistent with the Osborn et al. research, which revealed that in countries with high incomes, university mental health services are frequently underused (30). Notably, younger students in our study were more likely to identify instrumental challenges, possibly due to junior students’ lack of awareness about the university’s mental health facilities and their accessibility. These findings are in line with previous studies, which found that a majority of university students were uninformed about their institution’s mental health services (27, 31). Interestingly, students with a history of academic failure and those who had previously sought mental health services were more likely to experience stigma- and instrumental-related barriers. In line with earlier Saudi research which indicates that the primary factors behind the unmet need for mental health care in Saudi Arabia are more attitudinal than related to service availability or delivery (3).

Our study’s findings contribute significantly to the literature addressing the underutilisation of help-seeking services, potentially helping to mitigate the burden of mental disorders among medical students. The pattern of barriers to unmet treatment needs in this study was mainly students’ attitudes and stigma rather than the availability of mental health care, therefore university mental health service providers might benefit from taking specific steps towards this point, including providing students with comprehensive orientation sessions about available services and locations, organise workshops to enhance awareness and reduce stigma, as well as placing a greater emphasis on the roles of mentorship and counselling.

While this research stands as the first analysis of perceived needs and barriers concerning mental health service usage among Saudi medical students, it has certain limitations. First, its cross-sectional design restricts drawing causal conclusions. Second, the use of self-reported questionnaires could lead to recall bias. Third, the sensitivity of the subject matter could have influenced how participants responded to specific questions. Moreover, participants were only selected from the Eastern Province of Saudi Arabia, which may restrict the results’ generalizability. Lastly, the study does not explore in depth the issues related to students who begin but do not complete treatment, an area that should be addressed in future research.

## Conclusion

In conclusion, mental illnesses are prevalent among undergraduate medical students. Concurrently, while there is a pronounced perceived need for professional mental health support, actual service utilisation remains low. The primary obstacles to seeking mental care were found to be attitudinal and stigma-related barriers. Enhancing mental health literacy could play a pivotal role in addressing these unmet treatment requirements.

## Data availability statement

The original contributions presented in the study are included in the article/supplementary material, further inquiries can be directed to the corresponding author.

## Ethics statement

The study was approved by the Ethical Committee of King Faisal University, Al Ahsa, Saudi Arabia (Ref. No: KFU-REC-2022-MAY-ETHICS31; date: 24 May 2022). Additionally, participants received a brief description of the study’s purpose and a link to a non-personalised consent form.

## Author contributions

ZA: Conceptualization, Data curation, Formal analysis, Funding acquisition, Investigation, Methodology, Project administration, Resources, Software, Supervision, Validation, Visualization, Writing – original draft, Writing – review & editing. MS: Data curation, Formal analysis, Investigation, Methodology, Resources, Software, Validation, Writing – original draft, Writing – review & editing. AA-K: Data curation, Investigation, Writing – original draft, Writing – review & editing. JA: Data curation, Investigation, Writing – original draft, Writing – review & editing. RB: Data curation, Investigation, Writing – original draft, Writing – review & editing. MB: Data curation, Investigation, Writing – original draft, Writing – review & editing. RA: Data curation, Investigation, Writing – original draft, Writing – review & editing. NA-m: Data curation, Investigation, Writing – original draft, Writing – review & editing. MA: Data curation, Investigation, Writing – original draft, Writing – review & editing.

## References

[1] COVID-19 Mental Disorders Collaborators. Global prevalence and burden of depressive and anxiety disorders in 204 countries and territories in 2020 due to the COVID-19 pandemic. Lancet. (2021) 398:1700–12. doi: 10.1016/S0140-6736(21)02143-7 34634250 PMC8500697

[2] WHO (2023). Mental disorders. Available at: https://www.who.int/news-room/fact-sheets/detail/mental-disorders (Accessed August 26, 2023).

[3] Alangari AS Knox SS Kristjansson AL Wen S Innes KE Bilal L. Barriers to mental health treatment in the Saudi National Mental Health Survey. Int J Environ Res Public Health. (2020) 17:3877. doi: 10.3390/ijerph17113877, PMID: 32486182 PMC7311952

[4] Altwaijri YA Al-Habeeb A Al-Subaie AS Bilal L Al-Desouki M Shahab MK. Twelve-month prevalence and severity of mental disorders in the Saudi National Mental Health Survey. Int J Methods Psychiatr Res. (2020) 29:e1831. doi: 10.1002/mpr.1831 33245602 PMC7507007

[5] Al-Habeeb A Altwaijri YA Al-Subaie AS Bilal L Almeharish A Sampson NA. Twelve-month treatment of mental disorders in the Saudi National Mental Health Survey. Int J Methods Psychiatr Res. (2020) 29:e1832. doi: 10.1002/mpr.1832, PMID: 32519421 PMC7507396

[6] Almanasef M. Mental health literacy and help-seeking Behaviours among undergraduate pharmacy students in Abha. Saudi Arabia Risk Manag Healthcare Policy. (2021) 14:1281–6. doi: 10.2147/RMHP.S289211, PMID: 33790673 PMC8006949

[7] Zeng W Chen R Wang X Zhang Q Deng W. Prevalence of mental health problems among medical students in China: a meta-analysis. Medicine (Baltimore). (2019) 98:e15337. doi: 10.1097/MD.0000000000015337, PMID: 31045774 PMC6504335

[8] Rodriguez M Corse A Rosen L. Mental health services use among medical students: perceived stigma and barriers to care. Med Sci Educ. (2017) 27:267–72. doi: 10.1007/s40670-017-0392-6

[9] Hankir AK Northall A Zaman R. Stigma and mental health challenges in medical students. BMJ Case Rep. (2014) 2014:bcr2014205226. doi: 10.1136/bcr-2014-205226, PMID: 25183806 PMC4158203

[10] AlShamlan NA AlShamlan RA AlShamlan AA AlOmar RS AlAmer NA Darwish MA, et al. Prevalence of depression and its associated factors among clinical-year medical students in Eastern Province, Saudi Arabia. Postgrad Med J (2020) 96: 343–348. doi: 10.1136/postgradmedj-2020-137578, PMID: 32303582

[11] Bruffaerts R Mortier P Kiekens G Auerbach RP Cuijpers P Demyttenaere K. Mental health problems in college freshmen: prevalence and academic functioning. J Affect Disord. (2018) 225:97–103. doi: 10.1016/j.jad.2017.07.044, PMID: 28802728 PMC5846318

[12] Alaqeel M Alkhudairy FA Basuliman AS Alsubaie AM Alqahtani FN Almkainzi HA. The barriers to seeking mental health Services at King Saud bin Abdulaziz University for health sciences. Cureus. (2023) 15:e45321. doi: 10.7759/cureus.45321, PMID: 37849612 PMC10577391

[13] Givens JL Tjia J. Depressed medical students' use of mental health services and barriers to use. Acad Med. (2002) 77:918–21. doi: 10.1097/00001888-200209000-00024 12228091

[14] Noorwali R Almotairy S Akhder R Mahmoud G Sharif L Alasmee N. Barriers and facilitators to mental health help-seeking among young adults in Saudi Arabia: a qualitative study. Int J Environ Res Public Health. (2022) 19:2848. doi: 10.3390/ijerph19052848, PMID: 35270539 PMC8909985

[15] Alshahrani SS Alrajhi MM Alshehri MA Alotaibi FM Altwerqe MS. Challenges and barriers in primary mental health services in Saudi Arabia: a narrative review. Adv Hum Biol. (2023) 13:309–12. doi: 10.4103/aihb.aihb_54_23

[16] Baklola M Terra M Elzayat MA Abdelhady D El-Gilany AH Collaborators ATO. Pattern, barriers, and predictors of mental health care utilization among Egyptian undergraduates: a cross-sectional multi-Centre study. BMC Psychiatry. (2023) 23:139. doi: 10.1186/s12888-023-04624-z, PMID: 36879216 PMC9990190

[17] Kroenke K Spitzer RL Williams JB. The PHQ-9: validity of a brief depression severity. J Gen Intern Med. (2001) 16:606–13. doi: 10.1046/j.1525-1497.2001.016009606.x, PMID: 11556941 PMC1495268

[18] Adewuya AO Ola BA Afolabi OO. Validity of the patient health questionnaire (PHQ-9) as a screening tool for depression amongst Nigerian university students. J Affect Disord. (2006) 96:89–93. doi: 10.1016/j.jad.2006.05.021, PMID: 16857265

[19] Clement S Brohan E Jeffery D Henderson C Hatch SL Thornicroft G. Development and psychometric properties the barriers to access to care evaluation scale (BACE) related to people with mental ill health. BMC Psychiatry. (2012) 12:36. doi: 10.1186/1471-244X-12-36, PMID: 22546012 PMC3379935

[20] Rotenstein LS Ramos MA Torre M Segal JB Peluso MJ Guille C. Prevalence of depression, depressive symptoms, and suicidal ideation among medical students: a systematic review and Meta-analysis. JAMA. (2016) 316:2214–36. doi: 10.1001/jama.2016.17324, PMID: 27923088 PMC5613659

[21] Kihumuro RB Kaggwa MM Nakandi RM Kintu TM Muwanga DR Muganzi DJ. Perspectives on mental health services for medical students at a Ugandan medical school. BMC Med Educ. (2022) 22:734. doi: 10.1186/s12909-022-03815-8 36284284 PMC9592876

[22] Ewid M Amal Y Billah SMB Kalou Y Zitoun OA Alnaser AR. Impact of the COVID-19 pandemic on the psychological status of undergraduate medical students in Saudi Arabia: a cross-sectional double-scale study. Medicine (Baltimore). (2023) 102:e33487. doi: 10.1097/MD.0000000000033487, PMID: 37026919 PMC10081927

[23] Abdel-Hady D Baklola M Terra M El-Gilany AH. Patterns and barriers of mental health service utilization among medical students: a cross-sectional study. Middle East Curr Psychiatry. (2022) 29:98. doi: 10.1186/s43045-022-00267-0

[24] Phillips MS Steelesmith DL Brock G Benedict J Muñoz J Fontanella CA. Mental health service utilization among medical students with a perceived need for care. Acad Psychiatry. (2022) 46:223–7. doi: 10.1007/s40596-021-01584-y, PMID: 35006590

[25] Sontag-Padilla L Woodbridge MW Mendelsohn J D'Amico EJ Osilla KC Jaycox LH. Factors affecting mental health service utilization among California public college and university students. Psychiatr Serv. (2016) 67:890–7. doi: 10.1176/appi.ps.201500307, PMID: 27032662

[26] Cadigan JM Lee CM Larimer ME. Young adult mental health: a prospective examination of service utilization, perceived unmet service needs, attitudes, and barriers to service use. Prev Sci. (2019) 20:366–76. doi: 10.1007/s11121-018-0875-8, PMID: 29411197 PMC6081266

[27] Negash A Khan MA Medhin G Wondimagegn D Araya M. Mental distress, perceived need, and barriers to receive professional mental health care among university students in Ethiopia. BMC Psychiatry. (2020) 20:187. doi: 10.1186/s12888-020-02602-3, PMID: 32334569 PMC7183586

[28] Andrade G Bedewy D Elamin ABA Abdelmonem KYA Teir HJ Alqaderi N. Attitudes towards mental health problems in a sample of United Arab Emirates’ residents. Middle East Curr Psychiatry. (2022) 29:88. doi: 10.1186/s43045-022-00255-4

[29] Wijeratne C Johnco C Draper B Earl J. Doctors' reporting of mental health stigma and barriers to help-seeking. Occup Med (Lond). (2021) 71:366–74. doi: 10.1093/occmed/kqab119, PMID: 34534344

[30] Osborn TG Li S Saunders R Fonagy P. University students' use of mental health services: a systematic review and meta-analysis. Int J Ment Heal Syst. (2022) 16:57. doi: 10.1186/s13033-022-00569-0, PMID: 36527036 PMC9758037

[31] Ryan G Marley I Still M Lyons Z Hood S. Use of mental-health services by Australian medical students: a cross-sectional survey. Australas Psychiatry. (2017) 25:407–10. doi: 10.1177/1039856217715990, PMID: 28675041

